# Comparative Evaluation of Machine Learning Models and Conventional Formulas for LDL Cholesterol Estimation

**DOI:** 10.3390/diagnostics16132031

**Published:** 2026-06-29

**Authors:** Bagnu Orhan, Levent Deniz, Cengiz Aydin, Ipek Deveci Kocakoc

**Affiliations:** 1 Department of Medical Biochemistry, University of Health Sciences, Istanbul Training and Research Hospital, 34098 Istanbul, Türkiye; levent.deniz33@gmail.com; 2 Department of Medical Biochemistry, University of Health Sciences, Kanuni Sultan Suleyman Training and Research Hospital, 34303 Istanbul, Türkiye; drcengiz_aydin@yahoo.com; 3 Department of Econometrics, Faculty of Economics and Administrative Sciences, Dokuz Eylul University, 35340 Izmir, Türkiye; ipek.deveci@deu.edu.tr

**Keywords:** low-density lipoprotein cholesterol (LDL-C), hypertriglyceridemia, machine learning, Friedewald formula, Martin formula, Sampson formula

## Abstract

**Background/Objectives**: This study aimed to develop and externally validate machine learning (ML) models for low-density lipoprotein cholesterol (LDL-C) estimation and compare their analytical and clinical performance with conventional formulas, particularly in individuals with elevated triglyceride (TG) levels. **Methods**: This retrospective study included 11,681 adults whose lipid profiles were retrieved using a laboratory information system. ML models (linear regression, random forest, support vector regression, and XGBoost) were developed using routine lipid parameters and evaluated using 10-fold cross-validation. Performance was assessed using the mean absolute error (MAE), root mean squared error (RMSE), bias, correlation, Bland–Altman agreement, and clinical classification according to LDL-C categories. Subgroup analyses were conducted across TG strata, with an emphasis on TG ≥ 400 mg/dL. **Results**: ML models generally demonstrated lower error and higher agreement with directly measured LDL-C levels than conventional formulas. XGBoost showed the best overall performance (MAE: 14.7 mg/dL; RMSE: 20.22 mg/dL; R^2^ = 0.780; r = 0.88) and the lowest deviation. The ML models also showed a higher clinical classification accuracy (up to 66%). Performance declined with increasing TG levels, particularly for conventional formulas, whereas ML models remained more stable, including patients with TG ≥ 400 mg/dL. External validation across independent cohorts and analytical platforms demonstrated stable performance of the XGBoost model and generally higher classification accuracy than conventional LDL-C estimation formulas. **Conclusions**: ML-based LDL-C estimation may represent a complementary alternative to conventional formulas, particularly in hypertriglyceridemic populations.

## 1. Introduction

Atherosclerotic cardiovascular disease (ASCVD) is the leading cause of mortality and morbidity worldwide. Low-density lipoprotein cholesterol (LDL-C) is a modifiable risk factor for ASCVD and constitutes the primary objective of lipid-lowering therapy. Contemporary clinical guidelines emphasize LDL-C-guided risk stratification and treatment intensification, underscoring the need for accurate and reliable LDL-C measurements in routine practice [1,2,3,4].

The reference standard method for LDL-C measurement is beta-quantification. However, it demands labor-intensive handling procedures, including precipitation, ultracentrifugation, and a large sample volume. Thus, beta quantification is inappropriate for routine laboratory practice [5]. Although direct homogeneous LDL-C assays are available, they may incur additional costs [6]. Consequently, many medical laboratories opt for equations to estimate LDL-C levels. However, the performance of these equations varies considerably, and in some cases, proves inadequate when evaluated in various contexts [7]. The most commonly used LDL-C estimation formulas, including the Friedewald, Martin–Hopkins, and Sampson equations, rely on fixed or semi-fixed assumptions regarding the relationship between triglycerides (TG) and very-low-density lipoprotein cholesterol (VLDL-C) [8,9,10].

These assumptions may not hold across diverse metabolic states. In particular, the accuracy of conventional formulas deteriorates in the presence of elevated TG concentrations, non-fasting samples, and dyslipidemia. Systematic underestimation of LDL-C levels under such circumstances can lead to inappropriate classification into lower cardiovascular risk categories and potentially delay or attenuate lipid-lowering interventions [4,11]. These limitations highlight the need for more flexible and data-driven approaches for LDL-C estimation.

Machine learning (ML) methods offer the ability to model complex, non-linear relationships among lipid parameters without relying on predefined assumptions. By leveraging routinely available measurements, such as total cholesterol (TC), TG, and high-density lipoprotein cholesterol (HDL-C), ML-based models can adapt to heterogeneous lipid patterns and may provide more robust LDL-C estimates across a wide range of metabolic conditions. Recent advances in ML have demonstrated improved performance in clinical prediction tasks, suggesting its potential advantages over traditional formula-based approaches. Applying ML techniques to LDL-C estimation may therefore improve analytical accuracy and reduce clinically meaningful misclassifications, particularly in populations with hypertriglyceridemia.

Given the well-documented limitations of conventional LDL-C formulas in the presence of hypertriglyceridemia, the present study aimed to evaluate LDL-C estimation performance in a real-world cohort enriched with hypertriglyceridemic individuals. Because direct LDL-C measurements are routinely performed in our laboratory primarily in patients with markedly elevated triglyceride concentrations, the resulting retrospective dataset contained a high proportion of such individuals. This provided an opportunity to assess model robustness under metabolically challenging conditions. Accordingly, the study population does not represent a general screening cohort but rather a targeted laboratory population relevant to high-risk clinical scenarios in which LDL-C estimation formulas are known to perform less reliably.

The primary aim of this study was to develop and externally validate ML-based models for LDL-C estimation using routinely reported lipid parameters and to compare their performance with established conventional formulas. We hypothesized that ML-based approaches would demonstrate improved agreement with directly measured LDL-C levels, provide clinically more accurate classification according to the European Society of Cardiology (ESC) LDL-C categories [1], and maintain a more stable performance, especially in hypertriglyceridemic subgroups.

## 2. Materials and Methods

### 2.1. Study Population

Ethical approval for this study was granted by the Clinical Research Ethics Committee of Istanbul Training and Research Hospital (approval number: 39, date: 23 January 2026). All phases of this study were designed in accordance with the principles of the Declaration of Helsinki. Patients aged ≥18 years who underwent routine lipid profile tests, including TC, TG, HDL-C, and direct LDL-C, at Kanuni Sultan Suleyman Training and Research Hospital between 1 June 2024 and 30 June 2025, were included in the study. Data were obtained retrospectively using a laboratory information system (LIS). Individuals under the age of 18 years; those with lipid profiles with missing TC, TG, HDL-C, or direct LDL-C results; and those whose samples were rejected were excluded from the study. If multiple lipid profile results were available for the same patient during the study period, only the first measurement was included in the dataset. After applying the exclusion criteria, lipid profile results from 11,681 patients were analyzed in this study.

Baseline characteristics of continuous variables were summarized using mean ± Standard Deviation (SD) for approximately symmetric distributions (|skewness| ≤ 1.0) and median (Interquartile Range - IQR) for skewed distributions (|skewness| > 1.0). TG were reported as median (IQR) given their established right-skewed distribution. Distributional assessment included skewness and Shapiro–Wilk test.

In routine clinical practice, LDL-C is directly measured using a direct LDL-C assay in all patients with TG levels ≥400 mg/dL, and the directly measured LDL-C value is reported. However, for TG levels <400 mg/dL, the Friedewald method is used to calculate the LDL-C result. Furthermore, direct LDL-C measurements may still be requested by clinicians even in patients with TG levels <400 mg/dL. Consequently, this study included patients whose direct LDL-C measurements were retrospectively documented in the LIS.

### 2.2. Lipid Parameters

TC, TG, HDL-C, and direct LDL-C levels were measured using an automated clinical chemistry analyzer Cobas c702 (Roche Diagnostics, Mannheim, Germany) at the Kanuni Sultan Suleyman Training and Research Hospital Biochemistry Laboratory. TC concentrations were measured using an enzymatic oxidase–peroxidase method, whereas TG levels were determined using an enzymatic colorimetric assay based on the glycerol phosphate oxidase–peroxidase principle. HDL-C levels were measured using a homogeneous enzymatic colorimetric method employing cholesterol esterase, cholesterol oxidase, and peroxidase. LDL-C was measured using a direct enzymatic colorimetric method, in which selective surfactants were used to solubilize only LDL particles. In this method, enzymatic reactions toward non-LDL lipoproteins are inhibited by specific surfactants and sugar compounds, allowing selective measurement of free cholesterol and cholesterol esters within LDL particles.

The analyzer remained stable throughout the study period. The instrument was maintained in accordance with the manufacturer’s recommendations, and its analytical stability was monitored over time. Calibration procedures were performed using the original reagents and calibrators provided by Roche Diagnostics (Roche Diagnostics, Mannheim, Germany). To ensure the reliability of the results, internal quality control materials at two concentration levels (low and high) were analyzed daily, and an external quality assessment was performed at monthly intervals. The within-lab coefficients of variation for TC, TG, HDL-C, and LDL-C assays were 2.26%, 3.77%, 2.46% and 2.21%, respectively, for control 1; and 2.08%, 3.43%, 2.73%, and 2.69% for control 2. Both control levels met the European Federation of Clinical Chemistry and Laboratory Medicine (EFLM) allowable coefficients of variation criterion for lipid parameters [12].

### 2.3. Conventional Formulas

#### 2.3.1. Friedewald Formula

The Friedewald equation estimates LDL-C by subtracting HDL-C and VLDL-C from TC, where VLDL-C is approximated as TG/5 (mg/dL). Although widely used owing to its simplicity, the Friedewald formula assumes a fixed TG-to-VLDL-C ratio and is known to lose accuracy in the presence of elevated TG levels, particularly when TG concentrations exceed 400 mg/dL [8].

#### 2.3.2. Sampson Formula

The Sampson equation was developed to improve LDL-C estimation across a broader range of TG concentrations by incorporating the nonlinear relationship between TG and VLDL-C. This formula aims to provide more accurate estimates in patients with moderate hypertriglyceridemia while retaining computational simplicity. Nevertheless, its performance may be affected in cases of severe dyslipidemia [10].

#### 2.3.3. Martin–Hopkins Formula

The Martin–Hopkins method estimates LDL-C levels using an adjustable TG-to-VLDL-C ratio derived from strata based on TG and non-HDL-C concentrations. By replacing the fixed divisor used in the Friedewald formula with a data-driven factor, this approach improves accuracy, particularly at low LDL-C levels. However, its performance depends on the applicability of predefined strata to the study population and may vary across cohorts (http://www.ldlcalculator.com, accessed on 3 May 2026) [9].

### 2.4. Machine Learning Models

#### 2.4.1. Linear Regression

A multivariable linear regression model was used as the baseline ML approach. This model captures linear relationships between lipid parameters and LDL-C and provides an interpretable reference for comparison with more flexible nonlinear models. Linear regression or related regression-based approaches have been included in previous LDL-C estimation studies as comparator models when evaluating ML methods [13,14,15,16]. Population-adapted linear regression equations have been shown to outperform the Friedewald formula in independent cohorts across different analytical platforms [17].

#### 2.4.2. Random Forest

Random Forest is an ensemble learning method based on multiple decision trees trained on bootstrap samples with random feature selection. This approach can model nonlinear relationships and interactions among lipid parameters and is relatively robust to noise. Random Forest has been evaluated in LDL-C estimation studies and has shown good predictive performance compared with conventional LDL-C estimation formulas in several datasets [13,14,18].

#### 2.4.3. Support Vector Regression

Support Vector Regression (SVR) with a radial basis function kernel was used to model nonlinear associations between input features and LDL-C. SVR aims to minimize prediction error within a defined margin while maintaining model generalizability, making it suitable for regression tasks with complex feature relationships. Previous LDL-C estimation studies have evaluated SVM/SVR alongside other machine learning methods and reported favorable regression performance compared with traditional formulas such as the Friedewald and Martin equations [15,18].

#### 2.4.4. XGBoost

Extreme Gradient Boosting (XGBoost) is a gradient boosting-based ensemble method that builds sequential decision trees optimized through gradient-based loss minimization. XGBoost was included because of its suitability for structured clinical data and its ability to capture nonlinear effects and feature interactions. Previous studies on LDL-C estimation and LDL-C-related clinical prediction have reported strong performance for XGBoost or gradient boosting-based models, although performance varies according to dataset characteristics and outcome definition [13,14,16,18,19].

### 2.5. Model Training and Validation

#### 2.5.1. Feature Set Definition

ML models were developed to estimate directly measured LDL-C, which was used as the reference outcome. The analysis was restricted to complete lipid panels with available TC, TG, HDL-C, and directly measured LDL-C levels, yielding a final dataset of 11,681 observations. The input feature set for all ML models consisted of routinely reported lipid parameters, specifically TC, TG, and HDL-C levels. Age and sex were not included in model development because the primary objective was to evaluate LDL-C estimation based exclusively on routinely reported lipid profile parameters that are universally available within LISs. Restricting the feature set to lipid measurements was intended to maximize model applicability and facilitate implementation across different laboratory settings without requiring access to additional clinical information. Input features (TC, TG, HDL-C) were used in their original units (mg/dL) without additional rescaling or normalization. Tree-based models and linear regression were trained on these raw values. For SVR, the RBF kernel used gamma = “scale”, whereby the kernel bandwidth is derived from training-fold feature variance at each refit.

#### 2.5.2. Cross-Validation Strategy

Model performance was assessed using 10-fold cross-validation implemented with a K-fold procedure (*n*_splits = 10, shuffle = true, random_state = 42). In each iteration, nine folds were used for model training, and the remaining fold was reserved for validation, ensuring that each observation contributed to validation exactly once. Performance metrics were calculated exclusively on the held-out validation fold for each split and subsequently summarized as the mean ± SD across all folds. To enable unbiased global performance visualization and subgroup analyses, out-of-fold predictions from all validation folds were aggregated, ensuring that no observation was evaluated using a model trained on the same observation.

#### 2.5.3. Hyperparameter Optimization

Explicit hyperparameter optimization procedures, such as grid search or randomized search, were not applied in the present analysis. Instead, all models were trained using predefined parameter settings selected to provide stable performance and to allow transparent and fair comparison across methods. Linear regression models were fitted using default settings, while Random Forest, SVR, and XGBoost models were trained with fixed hyperparameter configurations defined a priori. Linear regression was fitted using scikit-learn default settings. Random Forest regression was trained with 200 decision trees (n_estimators = 200, random_state = 42); all other Random Forest parameters were left at scikit-learn defaults. Support vector regression used a radial basis function kernel with C = 10.0, epsilon = 1.0, and gamma = “scale”. XGBoost regression was configured with n_estimators = 200, learning_rate = 0.15, max_depth = 3, subsample = 0.85, colsample_bytree = 0.85, reg_lambda = 1.0, random_state = 42, tree_method = “hist”, and objective = “reg:squarederror”. These settings were fixed a priori and applied identically in every cross-validation fold. This strategy was adopted to minimize overfitting risk and to maintain methodological consistency across models. The absence of nested cross-validation or automated hyperparameter tuning is acknowledged as a methodological limitation and represents an area for future refinement.

#### 2.5.4. Prevention of Data Leakage

Several measures were implemented to minimize the risk of data leakage. First, strict separation between the training and validation data was maintained within each cross-validation fold, and all performance metrics were computed exclusively on unseen validation data. All models were refit independently within each cross-validation fold using training-partition data only; validation-partition information did not influence model fitting or SVR kernel scaling. Second, the pooled predictions used for summary analyses and visualizations were generated from out-of-fold estimates rather than from in-sample fitted values. Third, the feature set was restricted to analytes that were available prior to LDL-C calculation, thereby preventing inadvertent incorporation of information derived from the target variable. Model selection was based solely on cross-validated performance. Following completion of cross-validation and comparative evaluation, the best-performing model was refitted on the full dataset for potential deployment. This final refitting step was conducted after performance estimation and did not influence reported validation results.

#### 2.5.5. Model Explainability

To address model interpretability, gain-based feature importance was examined for the final XGBoost model refit on the full development cohort after cross-validation-based algorithm selection. Importance scores reflect the mean improvement in the squared error objective when each lipid variable (TC, TG, HDL-C) is used for splitting in the ensemble; values were expressed as relative percentages summing to 100% (Supplementary Table S1). This analysis was restricted to the development dataset; external validation cohorts were not used for interpretability estimation.

### 2.6. Statistical Analysis

All data processing, statistical analyses, and ML procedures were performed using Python v. 3.14.0. The analysis pipeline was implemented using standard scientific and ML libraries, including NumPy (v.2.3.5) and pandas (v.2.3.3) for data manipulation; scikit-learn (v.1.7.2) for model development, cross-validation, and performance evaluation; and XGBoost (v.3.1.3) for gradient boosting-based modeling. Statistical calculations, error metrics, correlation analyses, and Bland–Altman agreement measures were derived within this environment to ensure reproducibility and consistency across models. Model performance was primarily evaluated using descriptive statistics derived from cross-validation, with results reported as mean ± SD across folds. Comparative performance was interpreted based on the magnitude and consistency of differences in predefined metrics, including the mean absolute error (MAE), root mean squared error (RMSE), bias, correlation coefficients, Bland–Altman limits of agreement (LoA), and clinical classification accuracy. The strength of the linear association between estimated and directly measured LDL-C values was evaluated using Pearson’s correlation coefficient (r). The coefficient of determination (R^2^) was additionally reported to quantify the proportion of variance in the measured LDL-C level explained by each estimation method.

Agreement between estimated and directly measured LDL-C levels was further evaluated using the Bland–Altman analysis. For each method, the difference between the estimated and measured LDL-C levels was plotted against the mean of the two values. The mean difference (bias) and 95% LoA, defined as bias ± 1.96 standard deviations of the differences, were calculated. Bland–Altman analyses were performed using pooled out-of-fold predictions to ensure unbiased agreement assessment. In addition to the overall analysis, Bland–Altman results were examined across TG subgroups to explore the stability of agreement under varying metabolic conditions. This approach enabled the identification of systematic trends, proportional bias, and widening of the LoA at higher LDL-C or TG concentrations.

To evaluate the clinical relevance beyond numerical accuracy, estimated LDL-C values were categorized according to the ESC LDL-C categories [1]. Participants were divided into subcategories based on direct LDL-C values (<55 mg/dL, 55–69 mg/dL, 70–99 mg/dL, 100–115 mg/dL, 116–189 mg/dL, and ≥190 mg/dL). ESC classification accuracy was defined as the proportion of cases correctly assigned to the same LDL-C category as the direct LDL-C method. Misclassifications were further analyzed according to direction, distinguishing underestimation (assignment to a lower-risk category) from overestimation (assignment to a higher-risk category), given their differing implications for cardiovascular risk stratification and treatment decisions.

**Figure 1:**
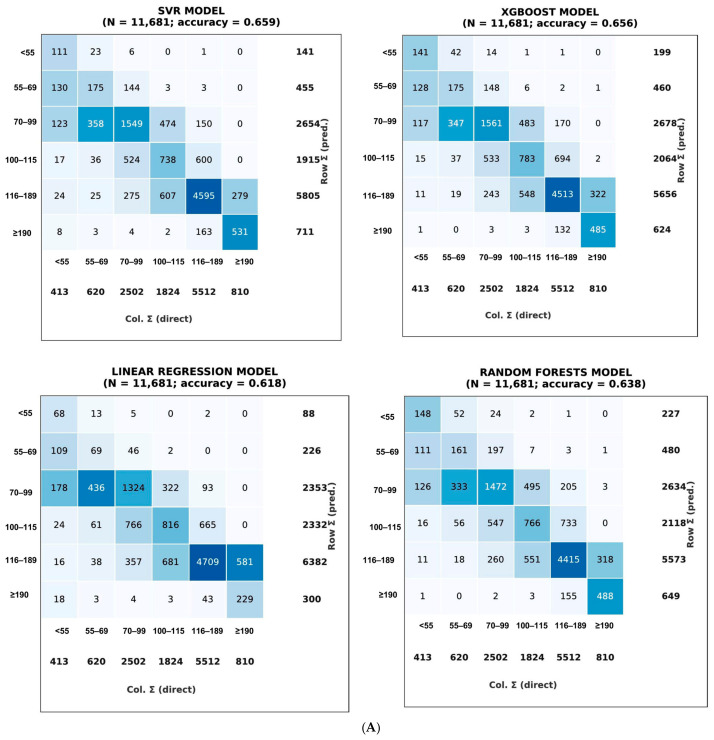

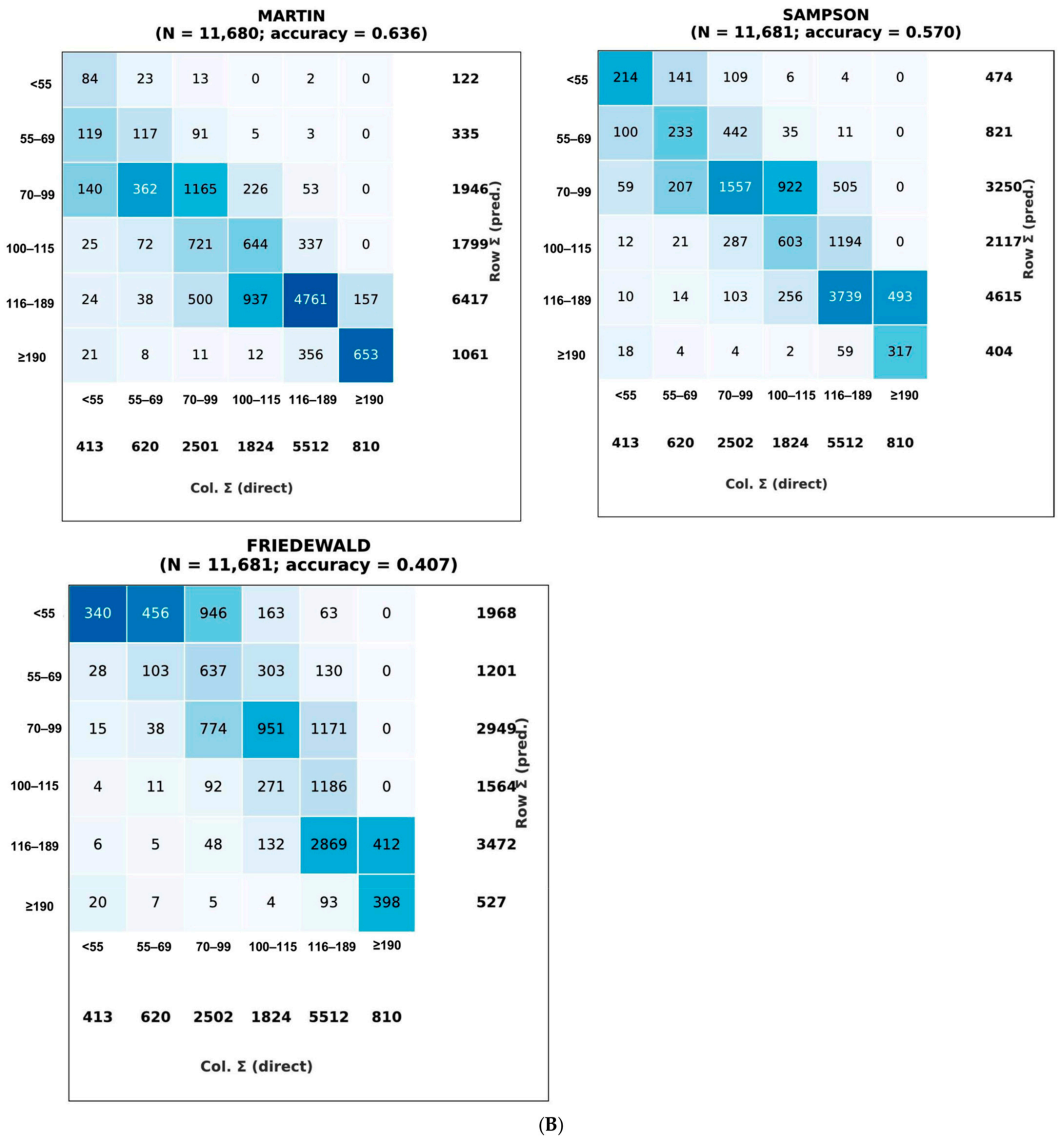
(**A**) Confusion matrices for machine learning–based LDL-C estimation methods (XGBoost, support vector regression, random forest, and linear regression) according to ESC LDL-C treatment categories.  (**B**) Confusion matrices for conventional LDL-C estimation formulas (Martin–Hopkins, Sampson, and Friedewald) according to ESC LDL-C treatment categories. Column totals represent directly measured LDL-C (reference); row totals represent predicted LDL-C. Cell shading reflects the column-wise proportion (%): within each direct LDL-C category (column), the intensity of blue indicates the percentage of patients assigned to each predicted category. Darker blue denotes a higher proportion (approaching 100%); lighter shades denote lower proportions. Values in each cell are absolute counts.

Because ESC LDL-C categories constitute a multiclass and imbalanced classification problem, overall accuracy was supplemented with additional metrics computed from pooled out-of-fold predictions across 10-fold cross-validation. Macro-averaged precision, recall, F1-score, and specificity were calculated as the unweighted mean of per-class values, thereby assigning equal weight to each ESC category regardless of its prevalence. Weighted-averaged precision, recall, and F1 were also reported, with each class contributing in proportion to its sample size. Micro-averaged F1 was included for completeness and is equivalent to overall accuracy in this setting. Per-class precision, recall, F1, and specificity were derived from confusion matrices (Figure 1). Macro-averaged one-vs-rest AUC was computed using continuous predicted LDL-C values; for each category, the score was defined as the negative absolute distance between the predicted LDL-C and the midpoint of that category interval. Accuracy is reported for consistency with prior studies but was not interpreted as the sole measure of clinical classification performance.

To evaluate the robustness and clinical applicability of LDL-C estimation methods under varying metabolic conditions, predefined subgroup analyses were conducted with a specific focus on TG concentration, given its well-known impact on the accuracy of conventional LDL-C formulas. As the performance of the current formulas varies at different TG levels, the test data were divided into four groups according to TG levels (<100, 100–199, 200–399, and ≥400 mg/dL). Within this high-TG subgroup, all performance metrics including error metrics, correlation coefficients, Bland–Altman agreement indices, and ESC LDL-C classification accuracy were recalculated using out-of-fold predictions. To further characterize the performance of the method across the full spectrum of TG concentrations, TG values were stratified into incremental ranges, extending from normotriglyceridemia to severe hypertriglyceridemia. Performance metrics were computed separately within each TG stratum to examine trends in analytical error, bias, correlation strength, and LoA as TG levels increased. The results from these analyses were used to identify TG thresholds at which clinically meaningful deviations emerged, thereby informing the potential applicability of LDL-C estimation methods in routine practice and in patients with marked dyslipidemia.

Bootstrap 95% confidence intervals for MAE, RMSE, and ESC classification accuracy were obtained from pooled out-of-fold predictions using 5000 patient-level resamples with replacement; pairwise MAE differences between models were tested with the Wilcoxon signed-rank test.

### 2.7. Performance Metrics

Analytical accuracy was assessed using MAE and RMSE, calculated as the absolute and squared differences, respectively, between estimated LDL-C values and directly measured LDL-C. MAE was used as the primary indicator of the average absolute deviation, whereas RMSE was included to reflect the influence of larger errors. The mean error (ME) was calculated to quantify systematic bias, with positive values indicating overestimation and negative values indicating underestimation. Percentage bias was computed as the mean relative difference between the estimated and measured LDL-C levels, expressed as a percentage of the measured value.

Pearson’s r was used to quantify the strength of linear association between estimated and directly measured LDL-C. R^2^ was calculated as R^2^ = 1 − Σ(y_i_ − ŷ_i_)^2^/Σ(y_i_ − ȳ)^2^. Unlike r^2^, this R^2^ penalizes systematic bias and may be near zero or negative when a method is poorly calibrated despite moderate correlation. Negative R^2^ indicates that predictions are less accurate than the sample mean. Since Pearson r and R^2^ measure different properties of the same data, their divergence is an expected finding; this is not a methodological inconsistency but rather a natural consequence of the systematic bias introduced by the Friedewald formula.

A Composite Overall Score was calculated to summarize performance across analytical and clinical dimensions. Seven metrics were included: MAE, RMSE, R^2^, Pearson r, absolute mean error, absolute percentage bias, and ESC classification accuracy. Each metric was converted to a score between 0 and 1 using min–max normalization across the seven methods in Table 1. For error-based metrics (MAE, RMSE, absolute ME , and absolute percentage bias), normalized score = 1 − (value − minimum)/(maximum − minimum). For metrics in which higher values indicate better performance (R^2^, r, and ESC accuracy), normalized score = (value − minimum)/(maximum − minimum). The Overall Score was defined as the unweighted arithmetic mean of the seven normalized scores (weight = 1/7 per metric). This index was used descriptively to summarize multidimensional performance and to select the ML algorithm refit on the full cohort. Primary interpretation was based on individual metrics in Table 1, not the composite score alone. Because min–max normalization depends on the comparator set, small absolute differences among similarly performing models may yield larger relative score separation.

## 3. Results

A total of 11,681 participants with available TC, TG, HDL-C, and LDL-C levels were included in the analysis. The baseline characteristics of the study population are summarized in Table 2. The mean age was 50.3 ± 12.7 years, and 60.2% of the participants were male. Lipid concentrations were 230 mg/dL (IQR 199–265) for TC, 476 mg/dL (IQR 425–570) for TG, 32.0 mg/dL (IQR 28.0–38.0) for HDL-C, 197 mg/dL (IQR 166–230) for non-HDL-C, and 123 ± 43.1 mg/dL (mean ± SD) for directly measured LDL-C. Remnant cholesterol was 72.0 mg/dL (IQR 56.0–92.0). The median TG/VLDL ratio was 6.77 (IQR 5.67–8.12).

Table 1 presents the comparative performances of the seven methods evaluated using 10-fold cross-validation. Among these approaches, the ML models, particularly XGBoost, SVR, and Random Forest, exhibited lower prediction errors, as reflected by reduced MAE and RMSE values, along with higher explanatory power indicated by higher R^2^ values. The correlation between directly measured LDL-C and XGBoost predictions was the highest among all evaluated approaches, exceeding that observed for other ML models, including SVR, random forest, and linear regression. In comparison, the conventional estimation formulas demonstrated weaker correlations with directly measured LDL-C values than the ML-based models. The XGBoost model demonstrated the best overall agreement with directly measured LDL-C values, achieving the most favorable performance across MAE, RMSE, R^2^, and bias. Linear regression yielded the lowest mean bias among the evaluated methods. Clinical classification accuracy was also generally higher for ML-based methods, although all approaches exhibited some degree of misclassification. All formula-based approaches showed substantially higher bias and wider Bland–Altman LoAs than the ML models. Notably, the Friedewald formula exhibited the poorest performance, with the highest MAE and RMSE values (Table 1). 

Bootstrap 95% confidence intervals from pooled out-of-fold predictions (5000 resamples) and paired MAE comparisons among the top three ML models are reported in Supplementary Table S3. Confidence intervals for MAE and RMSE overlapped substantially between XGBoost and SVR, and paired testing indicated only a small difference between these two methods (mean MAE difference: −0.129 mg/dL, 95% CI: −0.276 to 0.004; Wilcoxon *p* = 0.010). In contrast, Random Forest showed significantly higher MAE than both XGBoost (mean MAE difference: −0.859 mg/dL, 95% CI: −0.990 to −0.726; *p* < 0.001) and SVR (mean MAE difference: −0.729 mg/dL, 95% CI: −0.915 to −0.543; *p* < 0.001). Accordingly, the leading ML algorithms demonstrated broadly comparable performance, with SVR marginally superior for ESC classification and XGBoost for regression metrics; differences between XGBoost and SVR are unlikely to be clinically meaningful in this large cohort. All ML models nonetheless substantially outperformed conventional formulas, particularly Friedewald (Table 1; Supplementary Tables S2 and S3).

**Figure 2:**
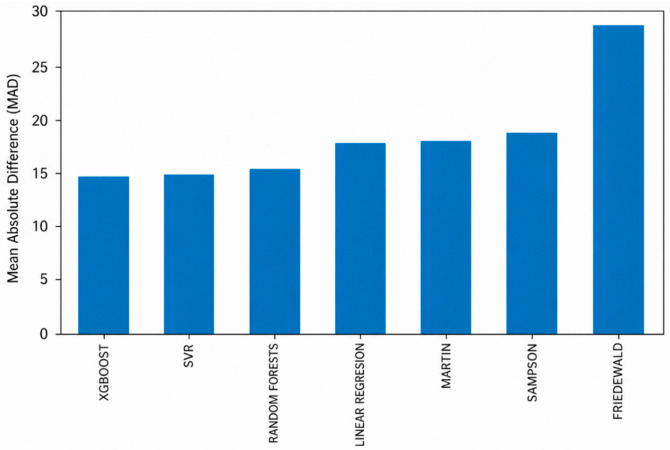
Comparison of mean absolute difference across machine learning models and conventional LDL-C estimation methods.

When the methods were ranked via an “Overall Score” that combined all metrics, ML models took the top four spots. The overall score represents a composite index derived from normalized analytical, agreement, and clinical classification metrics, with equal weighting applied to each component. XGBoost ranked first, with a score of 0.998 (Table 3). SVR and Random Forest showed closely similar composite scores (0.955) and nearly identical raw performance (MAE 14.81 and 15.54 mg/dL; r ≈ 0.87). The three ML models therefore demonstrated comparable overall accuracy. Mean Absolute Difference (MAD) indicates how much the predicted values deviate, on average, from the actual values. In the present analysis, ML-based models consistently demonstrated lower MAD values than conventional LDL-C estimation formulas. Among all the approaches evaluated, the XGBoost model exhibited the lowest MAD, which means a higher accuracy (MAD = 14.7), followed closely by SVR (14.8) and random forest (15.5). Linear regression analysis showed a comparatively high deviation (17.8). In contrast, formula-based methods yielded larger deviations, with MAD values of 17.9 for the Martin formula, 18.8 for the Sampson formula, and 28.9 for the Friedewald formula (Figure 2).

The clinical usefulness of a method depends not only on its numerical accuracy but also on its ability to classify patients into the correct treatment risk groups. In this analysis, the methods were evaluated in terms of their classification performance according to the LDL-C treatment targets defined by the ESC. The ESC category distribution was imbalanced, with 47.2% of participants in the 116–189 mg/dL category and 3.5% in the <55 mg/dL category (Supplementary Table S2). Therefore, macro-averaged classification metrics were evaluated in addition to overall accuracy. Clinical risk classification accuracy was highest among ML models, with SVR achieving the greatest overall accuracy (0.659), followed by XGBoost (0.656) and random forest (0.638). Under macro-averaged F1, which assigns equal weight to each category, XGBoost performed best (0.546), followed by SVR (0.539) and random forest (0.530); the top three ML models therefore showed closely similar extended classification performance. In contrast, Friedewald demonstrated substantially poorer multiclass performance (accuracy 0.407; macro-F1 0.346). Per-class precision, recall, and misclassification patterns are shown in Figure 1 and Supplementary Table S2. Among the conventional approaches, the Martin formula showed a comparable overall accuracy (64%) and notably the lowest rate of misclassification into lower-risk categories (8%), suggesting a reduced risk of undertreatment.

**Figure 3:**
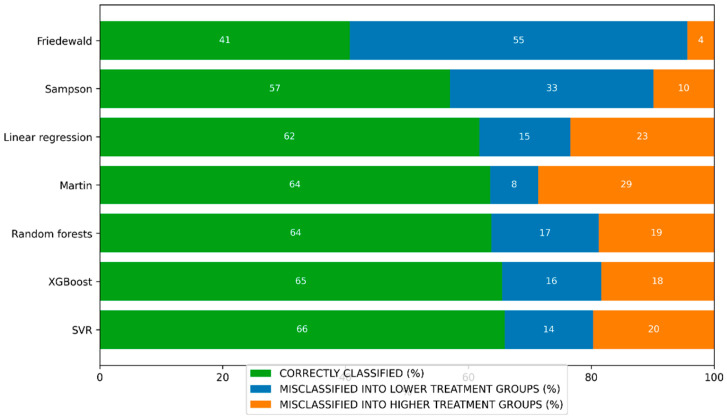
Comparison of clinical risk classification performance of LDL-C estimation methods.

However, this was accompanied by a relatively high rate of misclassification into high-risk categories (29%), which may lead to potential overtreatment. In contrast, the Sampson formula demonstrated a substantial tendency to misclassify patients into lower-risk categories (33%), raising concerns about possible undertreatment. Linear regression showed intermediate performance (62%), with moderate rates of both under- and overestimation. The Friedewald formula showed the poorest performance by misclassifying more than half of the patients (55%) into lower-risk categories. (Figure 3 and Figure 1). 

**Figure 4:**
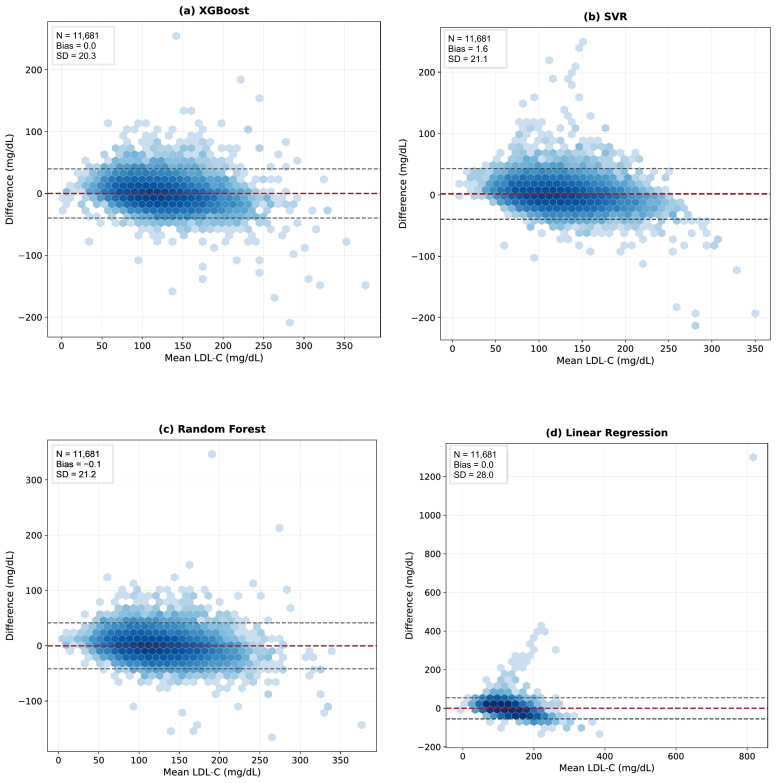

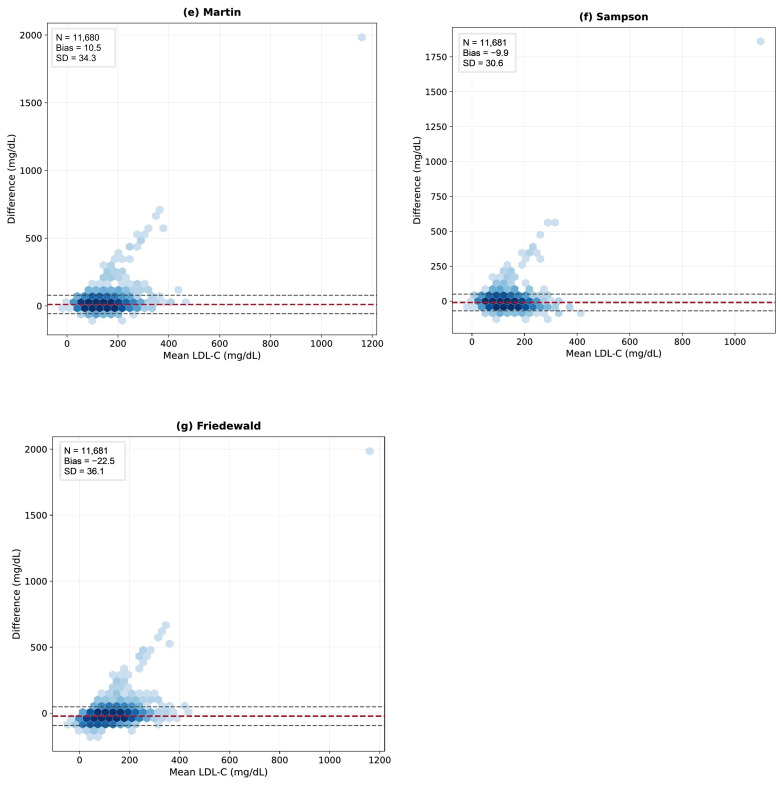
Bland–Altman plots comparing LDL-C estimates generated by machine learning models with directly measured LDL-C values. (**a**–**d**) correspond to the XGBoost , SVR, Random Forest, Linear regression models, respectively. Bland–Altman plots comparing LDL-C estimates generated by conventional formulas with directly measured LDL-C values. (**e**–**g**) correspond to the Martin–Hopkins, Sampson, and Friedewald formulas, respectively. In each panel, the x-axis is mean LDL-C (mg/dL) and the y-axis is the estimation difference (estimated − measured, mg/dL). Red and grey dashed lines denote mean bias and 95% limits of agreement, respectively. Darker blue indicates higher local point density (log scale, common across panels).

Bland–Altman analysis was used to evaluate the agreement between the predicted and directly measured LDL-C values. Figure 4 shows that ML models have both lower systematic deviations and tighter fit limits than the traditional formulas. XGBoost showed the best fit, with almost zero deviation (0.03 mg/dL) and the narrowest range of fit (79.29 mg/dL). In contrast, the Friedewald formula tends to systematically underestimate LDL-C with both a high negative deviation (−22.46 mg/dL) and the widest range of fit (141.69 mg/dL), indicating that its estimates are highly variable (Figure 4).

Performance across predefined TG subgroups is summarized in Table 4. ML models maintained low percentage bias across all TG ranges, whereas conventional formulas showed greater variability and larger errors, particularly at elevated TG levels. The relative advantage of individual methods shifted across subgroups: When evaluated according to percentage bias, SVR performed best at TG < 100 mg/dL, XGBoost at 100–199 and ≥400 mg/dL, and linear regression at 200–399 mg/dL. Among formulas, Martin–Hopkins was most accurate in the 200–399 mg/dL range, whereas Sampson was relatively better at very high TG levels. Across TG subgroups stratified in 100 mg/dL increments, the XGBoost model consistently demonstrated bias percentages below the predefined optimal bias threshold of 2.80% in all subgroups (Supplementary Table S5).

Supplementary Table S5 examines in detail the performance of the methods in subgroups with TG levels >400 mg/dL. As the TG levels increased, the performance of all methods tended to decrease. However, this decrease is much more pronounced in traditional formulas. When TG levels were examined in increments of 100 mg/dL, ML models (especially XGBoost and Random Forest) were compared. The XGBoost and Random Forest models were found to maintain a higher correlation than the other formulas. Analyses demonstrated consistently superior performance of ML models compared with conventional formulas at high TG ranges. Across TG subgroups stratified in 100 mg/dL increments, the XGBoost model consistently demonstrated bias percentages below the predefined optimal bias threshold of 2.80% in all subgroups (Supplementary Table S5) [12].

Although machine learning models are often regarded as less transparent than fixed formulas, feature importance analysis of the selected XGBoost model showed that LDL-C estimation was driven predominantly by TC (70.9% relative gain-based importance), with additional contributions from HDL-C (15.3%) and TGs (13.8%) (Supplementary Table S1). This pattern is biologically plausible because LDL-C is mathematically and clinically linked to the balance among total cholesterol, HDL-C, and triglyceride-rich lipoprotein-related information. While SHAP or partial dependence analyses could provide patient-level explanations, the present three-feature model is already directly interpretable in lipid biology terms; feature importance therefore supports clinical transparency without altering the primary performance conclusions.

### External Validation in Independent Hospital Cohorts

To assess the generalizability and transportability of the proposed model beyond the development dataset, external validation was performed in three independent hospital cohorts measured on different analytical platforms: Roche Cobas c702 (Roche Diagnostics, Mannheim, Germany) (*n* = 2957), Beckman Coulter (Beckman Coulter Inc., Brea, CA, USA) (*n* = 3986), and Siemens Atellica (Siemens Healthineers, Erlangen, Germany) (*n* = 18,244). All cohorts provided complete lipid profiles (TC, TG, HDL-C, and direct LDL-C) and were entirely independent of the original training dataset. Baseline demographic and biochemical characteristics of the development and external cohorts are summarized in Supplementary Table S6 to allow assessment of cohort comparability and potential distributional shifts between datasets. The XGBoost model trained on the development cohort was applied without retraining to each external dataset. For Beckman and Siemens, analyzer-specific post hoc calibration (70% fit/30% test split) was additionally evaluated using offset, slope–intercept, and triglyceride-conditioned quantile-regression adjustments.

Compared with the development cohort (Table 2; mean age 50.3 years; 60.2% male; mean LDL-C 123 mg/dL), the Roche validation cohort showed a younger mean age (46.2 years) but similar sex distribution (66.1% male) and broadly comparable lipid profiles (median TC 234 mg/dL [IQR 202–272], median TG 506 mg/dL [IQR 441–648], mean HDL-C 32.5 mg/dL, mean LDL-C 119 mg/dL), consistent with evaluation on the same analytical platform (Cobas c702). The Beckman cohort demonstrated a modestly older mean age (52.2 years) and a distributional shift toward higher direct LDL-C (mean 159 mg/dL) and HDL-C (median 44 mg/dL [IQR 38–52]) relative to the development cohort, reflecting both population and platform differences. The large Siemens cohort (*n* = 18,244) showed demographic and lipid distributions broadly similar to the development and Roche cohorts (mean age 47.1 years; median LDL-C 114 mg/dL [IQR 90–143]; median TG 494 mg/dL [IQR 437–614]), supporting evaluation of model transportability across centers and analytical systems.

In the Roche external validation cohort, the uncalibrated XGBoost model demonstrated preserved analytical performance (r = 0.84, R^2^ = 0.71, MAE = 17.8 mg/dL, RMSE = 23.7 mg/dL) with minimal systematic bias (mean error = −1.05 mg/dL; percent bias = −0.89%) and ESC category accuracy of 59.4%, exceeding Sampson (48.4%) and Friedewald (34.7%) on the same dataset (Table 5). In the Beckman cohort, uncorrected transport showed attenuated agreement (r = 0.82, MAE = 30.8 mg/dL) with a negative mean bias (−16.5%), reflecting platform-related offset. After device-specific TG-quantile regression calibration on the held-out 30% test subset, Beckman performance improved substantially (MAE = 8.70 mg/dL, R^2^ = 0.87, ESC accuracy = 87.0%). In the large Siemens cohort, the uncalibrated XGBoost model maintained acceptable agreement (r = 0.71, MAE = 16.9 mg/dL, percent bias = 2.07%, ESC accuracy = 61.9%) and outperformed Friedewald (38.5%) while performing comparably to Sampson for absolute error metrics. Slope–intercept calibration provided modest additional improvement on the Siemens test subset (MAE = 15.2 mg/dL). Collectively, these findings support cross-center transportability of the ML model, with platform-specific recalibration further improving performance where systematic device bias is present.

## 4. Discussion

In this study, we demonstrated that machine-learning-based models, particularly the XGBoost algorithm, offer a significant advantage over traditional formulas (Friedewald, Martin–Hopkins, and Sampson) in predicting LDL-C levels using routine lipid parameters. The XGBoost model demonstrated lower analytical error and better overall performance than conventional formulas, even in a high-risk cohort with higher TG levels (400 mg/dL), where the analytical performance of traditional formulas is known to be substantially reduced. Notably, across TG subgroups stratified in 100 mg/dL increments, the XGBoost model consistently maintained bias values below the predefined optimal deviation threshold of 2.80% in all subgroups (Supplementary Table S5). These findings suggest that the performance of conventional LDL-C estimation formulas varies across TG ranges, with each formula demonstrating optimal accuracy within specific subgroups.

In our study, although formula-based methods exhibited a higher bias than ML models, the Martin formula demonstrated lower MAD values than the Sampson and Friedewald formulas. The Martin formula demonstrated comparable overall accuracy and the lowest rate of misclassification into lower-risk categories, suggesting a reduced likelihood of underestimating patient risk and consequently minimizing missed treatment. However, this comes at the expense of a higher tendency to overestimate the risk, which may result in overtreatment in some patients. The present findings are consistent with those reported in the literature. Anudeep PP et al. reported that, among the conventional formulas evaluated in comparison with directly measured LDL-C, the Martin formula demonstrated the highest level of agreement (MAD = 9.19) [11]. Several studies have demonstrated that, particularly at elevated TG levels, the Martin formula showed better agreement with directly measured LDL-C levels than the Friedewald formula [20,21]. Meng JB et al. reported that conventional LDL-C estimation formulas, including the Friedewald equation, exhibit a significant decline in accuracy at TG levels >300 mg/dL. They further noted that the Sampson formula demonstrated superior predictive performance, particularly within the TG range of 200–300 mg/dL [14]. In our analysis; however, the Martin–Hopkins method demonstrated the best performance across the broader 200–399 mg/dL TG range, whereas the Sampson formula remained superior to the Friedewald equation. Previous studies have indicated that the concordance of the Sampson method was highest in patients with LDL-C below 70 mg/dL and TG levels above 400 mg/dL [10,22]. In our analysis, in the subgroup with TG levels ≥400 mg/dL, the Sampson formula demonstrated better agreement with directly measured LDL-C levels than other formula-based methods. Sajja et al. reported that the Sampson equation is more stable than the Friedewald equation at high TG levels but still contains significant error margins [23]. The Martin–Hopkins and Sampson equations were specifically developed to address the Friedewald limitations of elevated TG levels by introducing adjustable factors for the TG-to-VLDL-C ratio [9,10]. Our results confirm that these newer formulas show improved performance compared to Friedewald, which is consistent with previous studies. Azimi et al. reported that, for TG ≥ 400 mg/dL, the Martin–Hopkins and Sampson equations demonstrated reduced bias compared with the Friedewald equation, with the Martin–Hopkins equation showing lower misclassification rates than the Sampson equation [24]. Sajja et al. found that at TG levels of 400–799 mg/dL, the extended Martin–Hopkins equation was most accurate for LDL-C estimation (62.1%), while Friedewald (19.3%) and Sampson (40.4%) were less accurate [23]. Bayés et al. reported that for TG values 400–800 mg/dL, the Martin–Hopkins equation showed a 20.75 MAD and 30.48% misclassification, while Sampson showed a 31.36 MAD and 48.8% misclassification [25]. Consistent with our findings, Meeusen et al. found that the Martin formula may overestimate LDL-C levels in patients with high-risk ASCVD. The same study also showed that, when triglyceride concentrations exceed 400 mg/dL, the discontinuous table look-up structure of the formula could result in a shift from LDL-C underestimation to overestimation. Furthermore, in patients with triglyceride levels between 400 and 800 mg/dL, the Sampson formula demonstrated higher accuracy than the Martin formula [26].

This study suggested that ML approaches, particularly XGBoost, offer substantial and consistent improvements over conventional formula-based methods for LDL-C estimation across multiple performance dimensions. Our findings demonstrated that XGBoost achieved superior analytical accuracy (MAE: 14.7 mg/dL, RMSE: 20.22 mg/dL, R^2^: 0.780), highest correlation with directly measured LDL-C (r = 0.88), optimal agreement characteristics (bias: 0.03 mg/dL), and superior clinical classification accuracy (65%) compared to all evaluated methods. These results align with and extend the growing body of evidence supporting ML superiority in LDL-C estimation [13,14,16,27]. Kim et al. indicated that a 2-step ML model attained an RMSE of 7.015, in contrast to Friedewald (12.112), Martin–Hopkins (8.084), and Sampson (8.492), achieving an 85.1% concordance rate for LDL-C classification [13]. Similarly, Singh et al. showed that the Weill Cornell ML model had a correlation of 0.982 with direct LDL-C, which was better than that of Friedewald (0.950) and Martin–Hopkins (0.962) [28]. These findings are consistent with those of Çubukçu et al. and Meng et al., which highlight the success of ML models in modeling complex relationships between lipid parameters [14,27]. The limitation of the Friedewald formula to TG < 400 mg/dL and its systematic underestimation at high TG levels pose a significant clinical challenge [8]. The consistent pattern of Friedewald underestimation across multiple studies suggests a systematic bias that may contribute to widespread undertreatment of cardiovascular risk [29]. Our finding that the Friedewald formula misclassified 55% of patients into lower-risk categories suggests that formula-based estimation may contribute to this treatment gap. Our findings suggest that ML models may partially overcome this biochemical limitation and could represent a closer alternative to direct LDL-C measurement compared with conventional estimation formulas. XGBoost maintained bias below 2.80% even at TG levels ≥400 mg/dL, examined in fine-grained 100 mg/dL increments, demonstrates that ML approaches may reduce the need for direct LDL-C measurement, even in patients with hypertriglyceridemia. ML models, particularly XGBoost and SVR, demonstrated a more balanced distribution of misclassifications between the lower- and higher-risk categories, suggesting a potential advantage in reducing both undertreatment and overtreatment. Traditional formulas are based on the assumption that the VLDL-C/TG ratio is constant. The mechanistic basis for ML superiority lies in the ability of ensemble methods, such as XGBoost, to capture complex, non-linear relationships between lipid components without requiring explicit mathematical formulation of biochemical relationships [30]. Paplomatas et al. reported that an integrated ML approach combining 13 existing estimation equations with patient data achieved an R^2^ exceeding 0.98, significantly outperforming models relying solely on basic clinical parameters (R^2^ of approximately 0.95) [30]. This suggests that ML models learn intricate interactions and non-linearities that are not adequately represented by the simplified assumptions underlying conventional formulas, such as the fixed TG-to-VLDL-C ratio of 5:1 assumed by the Friedewald equation [8,11]. In our study, even at an median TG level of 476 mg/dL (IQR 425–570), the XGBoost model demonstrated superior robustness compared to the Sampson and Martin–Hopkins equations. Notably, linear regression, despite being an ML–based approach, showed only moderate classification accuracy (62%) and remained inferior to the Martin formula, which achieved a comparable overall accuracy of 64% among conventional methods. These findings further suggest that advanced ML algorithms, particularly ensemble methods, may better capture the complex effects of elevated TG levels on LDL-C than traditional formulas and simpler linear models. Despite the higher performance of ML models compared with conventional formulas, several limitations should be considered when interpreting these findings. Although analytical errors and bias were reduced, residual prediction errors remained present, and the Bland–Altman limits of agreement indicated substantial variability at the individual-patient level. Furthermore, clinical classification accuracy remained moderate, with approximately one-third of patients still being assigned to an incorrect LDL-C category. Finally, prospective studies evaluating the impact of ML-based LDL-C estimation on clinical decision-making and patient outcomes are needed before broader clinical implementation can be considered.

A recent large-scale study by Győrfi et al. developed platform- and population-specific LDL-C estimation equations using linear regression models in two independent cohorts comprising 31,265 individuals, including 10,174 samples analyzed on Roche Cobas systems. Their platform-specific equations showed markedly improved analytical and clinical performance compared with the Friedewald equation, with near-zero median errors and overall clinical classification accuracies of 85.1% for the Roche Cobas cohort and 78.6% for the Abbott Alinity cohort, compared with 67.1% and 65.3% for the Friedewald equation, respectively. These findings suggest that tailoring LDL-C estimation models to specific analytical platforms and study populations may improve estimation performance compared with the application of universally derived equations. Methodologically, the study shares several characteristics with the present investigation, including the use of large real-world datasets, Roche analytical platforms, and data-driven approaches based on routinely available lipid parameters. However, an important distinction lies in the composition of the study populations. While Győrfi et al. developed their equations in a broadly representative laboratory cohort, the present study was specifically designed to assess LDL-C estimation in a triglyceride-enriched population. Of the 11,681 individuals included in our analysis, 10,738 (91.9%) had TG concentrations ≥400 mg/dL, whereas only approximately 2.5% of samples in the study by Győrfi et al. exceeded this threshold. Given the well-recognized deterioration in LDL-C estimation accuracy at high TG concentrations, direct comparison of performance metrics between the two studies should therefore be interpreted with caution. Although the platform-specific regression equations reported by Győrfi et al. achieved higher overall classification accuracies (85.1%) than the best-performing model in our study (XGBoost, 65%), their cohort contained relatively few individuals with severe hypertriglyceridemia. In contrast, our study specifically focused on a population in which extreme TG concentrations were highly prevalent, representing a substantially more challenging setting for LDL-C estimation. Collectively, the findings of both studies indicate that data-driven LDL-C estimation approaches can improve agreement with directly measured LDL-C values, although their relative performance may depend on the characteristics of the target population and the prevalence of severe hypertriglyceridemia [17].

Zararsız et al. stated that the performance of formulas may vary on different laboratory platforms [22]. A key requirement for clinical adoption of machine learning models is successful external validation in cohorts independent of the training dataset. In the present study, external validation was therefore extended to three different analytical platforms: Roche Cobas c702 (*n* = 2957), Beckman Coulter AU5800 (*n* = 3986), and Siemens Atellica CH930 (*n* = 18,244) (Supplementary Table S6). In the Roche cohort, which used the same analytical system as the development dataset, the uncalibrated XGBoost model demonstrated preserved performance (r = 0.84; MAE = 17.8 mg/dL) with minimal systematic bias (mean error = −1.05 mg/dL; percent bias = −0.89%), remaining within the predefined optimal bias threshold of 2.80% [12]. In the large Siemens cohort, absolute error remained acceptable without model retraining (MAE = 16.9 mg/dL; percent bias = 2.07%; ESC accuracy = 61.9%), although correlation was modestly attenuated compared with the development and Roche cohorts (r = 0.71). In contrast, uncorrected transport to the Beckman platform showed poorer agreement (MAE = 30.8 mg/dL; percent bias = −16.5%), consistent with platform-related calibration differences. This finding aligns with Soussi et al., who noted that models often exhibit poorer calibration upon external validation and that inter-laboratory variability in biomarker assays may affect generalizability [31]. In our study, device-specific post hoc recalibration substantially improved Beckman performance on the held-out test subset (MAE = 8.70 mg/dL; ESC accuracy = 87.0%), whereas slope–intercept calibration provided only modest additional benefit on Siemens (MAE = 15.2 mg/dL). Across all external cohorts, XGBoost outperformed Friedewald and remained competitive with or superior to Sampson for regression-based metrics (Table 5). Collectively, these results indicate that XGBoost transportability is platform-dependent: performance is well preserved on the training analytical ecosystem and remains clinically useful on an independent large Siemens cohort, whereas deployment on a different platform such as Beckman may require local recalibration before clinical use.

This study had several notable strengths. First, it is based on a large dataset comprising complete lipid profiles, which enables robust model development and reliable internal validation. Second, the performance evaluation was comprehensive, integrating analytical error metrics, Bland–Altman agreement analysis, and clinically relevant classification according to ESC LDL-C risk categories. Third, a cross-validation framework with out-of-fold prediction aggregation was employed to minimize optimistic bias and ensure unbiased performance estimation. Fourth, detailed TG-stratified subgroup analyses were conducted, allowing the systematic evaluation of model performance under conditions known to challenge LDL-C estimation. Finally, external validation was extended to three independent Roche, Beckman, and Siemens platforms (total *n* = 25,187), with baseline characteristics reported to assess cohort comparability (Supplementary Table S6).

This study had several limitations. First, although external validation was performed on Roche, Beckman, and Siemens platforms (Supplementary Table S6), transportability was platform-dependent: uncorrected XGBoost performance was well preserved on the development analytical system (Roche) and acceptable in the large Siemens cohort, but attenuated on Beckman and improved only after device-specific post hoc recalibration on a held-out test subset. Accordingly, findings cannot be assumed to generalize uniformly to all analytical platforms, local calibration schemes, or patient populations without site-specific evaluation. External validation was also limited to the selected XGBoost model; transportability of SVR and Random Forest was not assessed in independent cohorts. Second, hyperparameter optimization was not performed using nested cross-validation, which may have limited the maximum achievable performance of some models. Third, the reference LDL-C method, although directly measured, reflects routine clinical practice rather than a β-quantification gold standard, which may influence absolute agreement estimates. Although the TG-enriched design was intentional and reflected the primary objective of evaluating model performance in hypertriglyceridemic individuals, this imbalance may limit direct comparisons across triglyceride categories and restrict the generalizability of subgroup-specific findings. Finally, the models were developed exclusively using routinely reported lipid parameters (TC, TG, and HDL-C), whereas potentially relevant demographic and clinical variables such as age, sex, fasting status, diabetes mellitus, obesity, metabolic syndrome, lipid-lowering therapy, and other comorbid conditions were not available for model training. Although this approach enhances practical applicability and facilitates implementation within laboratory information systems, the omission of these variables may limit model personalization, robustness across specific patient subgroups, and transportability to populations with different clinical characteristics. Future studies incorporating both laboratory and clinical variables may further improve predictive performance and generalizability.

## 5. Conclusions

In conclusion, our study might represent a significant advance in the clinical application of artificial intelligence in laboratory medicine. We demonstrated that the XGBoost algorithm provided more accurate and stable LDL-C predictions than the Friedewald, Martin–Hopkins, and Sampson formulas, even in patients with severe hypertriglyceridemia. Although ML models demonstrated improved clinical classification performance, residual misclassification remained present. Consequently, direct LDL-C measurement may still be warranted in selected clinical scenarios, particularly when estimated LDL-C values lie near ESC treatment cut-offs or when precise risk stratification is required for therapeutic decision-making. The ability of ML models to maintain accuracy at high TG levels (≥400 mg/dL), where conventional formulas tend to perform less reliably, suggests that these approaches may serve as a helpful tool for LDL-C estimation in patients with hypertriglyceridemia. Although ML models demonstrate better performance, their complexity may limit their clinical implementation, thereby highlighting the need for their integration into LIS for routine use. Future multicenter and prospective studies are needed to confirm broader generalizability beyond the platforms evaluated here.

## Figures and Tables

**Table 1:** Comparative performance of machine learning and conventional LDL-C estimation methods.

Method	r	MAE mg/dL	RMSE mg/dL	R^2^	Mean Error mg/dL	Bias%	Accuracy	Mean Bias mg/dL	LoA mg/dL
XGBoost	0.88 ± 0.01	14.7 ± 0.44	20.2 ± 0.56	0.78 ± 0.02	0.03 ± 0.69	0.02 ± 0.56	0.656 ± 0.02	0.03 ± 0.69	79.3 ± 2.18
SVR	0.87 ± 0.01	14.8 ± 0.41	21.2 ± 0.77	0.76 ± 0.02	1.63 ± 0.69	1.32 ± 0.56	0.659 ± 0.02	1.63 ± 0.69	82.7 ± 3.03
RF	0.87 ± 0.01	15.5 ± 0.52	21.1 ± 1.01	0.76 ± 0.03	−0.12 ± 0.56	−0.10 ± 0.45	0.638 ± 0.02	−0.12 ± 0.56	82.9 ± 3.96
LR	0.79 ± 0.10	17.8 ± 0.57	27.1 ± 7.33	0.57 ± 0.31	0.03 ± 0.75	0.03 ± 0.61	0.618 ± 0.02	0.03 ± 0.75	106 ± 28.7
Martin	0.76 ± 0.12	17.9 ± 1.02	33.8 ± 12.9	0.29 ± 0.72	10.5 ± 1.06	8.51 ± 0.89	0.636 ± 0.02	10.5 ± 1.06	125 ± 51.5
Sampson	0.79 ± 0.13	18.8 ± 0.93	30.1 ± 11.9	0.43 ± 0.62	−9.91 ± 0.89	−8.03 ± 0.70	0.570 ± 0.02	−9.91 ± 0.89	110 ± 48.9
Friedewald	0.78 ± 0.11	28.9 ± 1.03	41.2 ± 11.3	0.01 ± 0.73	−22.5 ± 1.05	−18.2 ± 0.94	0.407 ± 0.01	−22.5 ± 1.04	133 ± 50.9

The table summarizes the performance of the seven methods based on the 10-fold cross-validation results. Metrics are reported as mean ± SD across folds. XGBoost, extreme gradient boosting; SVR, support vector regression; RF, random forest; LR, linear regression; MAE, mean absolute error; RMSE, root mean square error; LoA, limits of agreement. R^2^ mean ± SD for formula methods can be large because per-fold R^2^ crosses zero. ESC classification accuracy in Table 1 denotes overall (micro) accuracy. Macro-averaged precision, recall, F1, specificity, and multiclass AUC are provided in Supplementary Table S2. Bootstrap 95% confidence intervals for MAE, RMSE, and ESC classification accuracy are reported in Supplementary Table S3. Metric abbreviations and definitions are provided in Supplementary Table S4.

**Table 2:** Characteristics of the study population (complete-case *N* = 11,681).

Characteristic	Value
Sex—Male	7028 (60.2%)
Age (years)	50.3 ± 12.7
Total cholesterol (mg/dL)	230 (199–265)
Triglycerides (mg/dL)	476 (425–570)
HDL-C (mg/dL)	32.0 (28.0–38.0)
non-HDL-C (mg/dL)	197 (166–230)
LDL-C (mg/dL)	123 ± 43.1
Remnant cholesterol (mg/dL)	72.0 (56.0–92.0)
TG/VLDL ratio	6.77 (5.67–8.12)

Continuous variables were assessed for distributional symmetry using skewness. Variables with |skewness| > 1.0 and triglycerides (per lipid literature convention) are reported as median (interquartile range); other continuous variables as mean ± SD. Normality was additionally evaluated using Shapiro–Wilk tests. Sex is *n* (%). HDL-C, high-density lipoprotein cholesterol; non-HDL-C, non-high-density lipoprotein cholesterol; LDL-C, low-density lipoprotein cholesterol; TG/VLDL, triglyceride to very low-density lipoprotein cholesterol ratio.

**Table 3:** Overall Method Ranking (General Score).

Order	Method	Overall Score
1	Machine Learning—XGBoost	0.998
2	Machine Learning—SVR	0.955
3	Machine Learning—Random Forest	0.955
4	Machine Learning—Linear Regression	0.757
5	Formula—Sampson	0.544
6	Formula—Martin	0.495
7	Formula—Friedewald	0.025

Overall Score = unweighted mean of seven min–max normalized metrics across all methods in Table 1. Individual metrics should be used for clinical interpretation. The top three ML models showed similar MAE (14.7–15.5 mg/dL).

**Table 4:** Performance of LDL-C estimation methods across triglyceride ranges.

Method	Range	*N*	Mean ± SD mg/dL	MAE mg/dL	Mean Error mg/dL	Bias %	r
Direct LDL-C	<100 mg/dL	269	107.2 ± 32.22	NA	NA	NA	NA
Linear Regression	<100 mg/dL	269	132.8 ± 24.09	26.5	25.6	23.8%	0.86 *
Random Forest	<100 mg/dL	269	106.3 ± 31.08	5.28	−0.90	−0.84%	0.97 *
SVR	<100 mg/dL	269	107.9 ± 27.91	6.83	0.65	0.60%	0.95 *
XGBoost	<100 mg/dL	269	105.6 ± 30.13	5.49	−1.59	−1.48%	0.97 *
Friedewald	<100 mg/dL	269	102.4 ± 32.23	5.75	−4.83	−4.51%	0.96 *
Sampson	<100 mg/dL	269	104.9 ± 31.35	4.63	−2.34	−2.18%	0.96 *
Martin	<100 mg/dL	269	101.0 ± 31.15	6.75	−6.22	−5.80%	0.98 *
Direct LDL-C	100–199 mg/dL	297	130.3 ± 40.53	NA	NA	NA	NA
Linear Regression	100–199 mg/dL	297	142.8 ± 83.42	21.0	12.4	9.53%	0.32
Random Forest	100–199 mg/dL	297	131.1 ± 40.89	7.75	0.81	0.62%	0.92 *
SVR	100–199 mg/dL	297	128.0 ± 36.38	8.05	−2.32	−1.78%	0.93 *
XGBoost	100–199 mg/dL	297	130.7 ± 39.03	7.38	0.31	0.24%	0.94 *
Friedewald	100–199 mg/dL	297	129.4 ± 127.2	19.2	−0.91	−0.70%	0.32
Sampson	100–199 mg/dL	297	132.5 ± 119.2	16.1	2.12	1.62%	0.32
Martin	100–199 mg/dL	297	131.9 ± 126.5	17.2	1.64	1.25%	0.31
Direct LDL-C	200–399 mg/dL	377	138.4 ± 43.25	NA	NA	NA	NA
Linear Regression	200–399 mg/dL	377	138.4 ± 29.11	15.1	0.02	0.01%	0.93 *
Random Forest	200–399 mg/dL	377	138.8 ± 41.26	8.98	0.45	0.32%	0.95 *
SVR	200–399 mg/dL	377	141.4 ± 41.27	8.41	3.03	2.19%	0.96 *
XGBoost	200–399 mg/dL	377	138.6 ± 40.67	8.47	0.22	0.16%	0.96 *
Friedewald	200–399 mg/dL	377	127.4 ± 44.92	13.4	−11.0	−7.96%	0.96 *
Sampson	200–399 mg/dL	377	132.5 ± 39.55	9.86	−5.89	−4.26%	0.96 *
Martin	200–399 mg/dL	377	139.2 ± 41.09	8.41	0.80	0.58%	0.96 *
Direct LDL-C	≥400 mg/dL	10,738	123.1 ± 43.26	NA	NA	NA	NA
Linear Regression	≥400 mg/dL	10,738	122.2 ± 31.90	17.6	−0.95	−0.77%	0.82 *
Random Forest	≥400 mg/dL	10,738	122.9 ± 38.77	16.2	−0.15	−0.12%	0.87 *
SVR	≥400 mg/dL	10,738	124.8 ± 38.11	15.4	1.71	1.39%	0.87 *
XGBoost	≥400 mg/dL	10,738	123.2 ± 38.33	15.3	0.06	0.05%	0.88 *
Friedewald	≥400 mg/dL	10,738	99.22 ± 52.28	30.3	−23.9	−19.4%	0.80 *
Sampson	≥400 mg/dL	10,738	112.5 ± 39.16	19.6	−10.6	−8.59%	0.81 *
Martin	≥400 mg/dL	10,738	134.6 ± 44.85	18.6	11.5	9.34%	0.78 *

*N* = number of samples within the triglyceride range (from combined out-of-fold predictions). * *p* < 0.001. Subgroup SD reflects dispersion of predicted values and may be inflated by formula-generated outliers in extreme lipid profiles.

**Table 5:** External validation performance of the XGBoost model compared with the Sampson and Friedewald formulas across three independent hospital cohorts (uncalibrated XGBoost).

Model	Roche			Beckman			Siemens		
	XGBoost	Sampson	Friedewald	XGBoost	Sampson	Friedewald	XGBoost	Sampson	Friedewald
*N*	2957	2957	2957	3986	3986	3986	18,244	18,244	18,244
r	0.84	0.76	0.73	0.82	0.86	0.79	0.71	0.73	0.67
MAE (mg/dL)	17.8	23.0	41.0	30.8	51.1	69.5	16.9	18.4	33.9
RMSE (mg/dL)	23.7	32.8	58.0	35.8	54.0	79.6	33.7	34.1	55.5
R^2^	0.71	0.44	−0.75	0.23	−0.75	−2.80	0.49	0.47	−0.40
Mean Error (mg/dL)	−1.05	−13.8	−34.8	−26.2	−49.4	−67.2	2.46	−10.1	−28.9
Bias (%)	−0.89	−11.6	−29.3	−16.5	−31.1	−42.2	2.07	−8.48	−24.4
ESC Accuracy	59.4%	48.4%	34.7%	50.2%	23.6%	17.5%	61.9%	56.8%	38.5%
BA Mean Bias (mg/dL)	−1.05	−13.8	−34.8	−26.2	−49.4	−67.2	2.46	−10.1	−28.9
BA SD of Differences (mg/dL)	23.7	29.7	46.4	24.4	21.8	42.7	33.6	32.5	47.4
BA Lower LoA (mg/dL)	−47.5	−72.0	−126	−74.0	−92.2	−151	−63.4	−73.8	−122
BA Upper LoA (mg/dL)	45.4	44.4	56.1	21.6	−6.6	16.6	68.3	53.7	64.0
BA LoA Width (mg/dL)	92.8	116	182	95.6	85.6	167	132	128	186

All metrics were computed on the full external cohort without model retraining. ESC accuracy denotes overall (micro) classification accuracy across ESC LDL-C treatment categories. BA, Bland–Altman; LoA, limits of agreement.

## Data Availability

The original contributions presented in this study are included in the article. Further inquiries can be directed to the corresponding author.

## Supplementary Materials

The following supporting information can be downloaded at: https://www.mdpi.com/article/10.3390/diagnostics16132031/s1, Table S1: Gain-based feature importance of the XGBoost model for total cholesterol, triglycerides, and HDL-C in the development cohort (*N* = 11,681); Table S2: ESC multiclass classification metrics for LDL-C estimation methods; Table S3: Bootstrap 95% confidence intervals for MAE, RMSE, and ESC classification accuracy based on pooled out-of-fold predictions from 10-fold cross-validation (development cohort, *N* = 11,681); Table S4: Mathematical definitions of evaluation metrics; Table S5: Comparative Performance of LDL-C Estimation Methods at High Triglyceride Levels (>400 mg/dL); Table S6: Baseline demographic and biochemical characteristics of the development and external validation cohorts.
