# Supplementary material for: Comparative Evaluation of Machine Learning Models and Conventional Formulas for LDL Cholesterol Estimation
Source: Diagnostics (Basel). 2026 Jun 29;16(13):2031. doi: 10.3390/diagnostics16132031 (PMC13359852; doi:10.3390/diagnostics16132031)
Supplement: Supplementary file 1 [file diagnostics-16-02031-s001.zip › diagnostics-4332254-supplementary.pdf]

Table S1. Gain-based feature importance of the XGBoost model for total cholesterol, triglycerides, and HDL-C in the development cohort (N = 11,681).

Feature	gain	weight	cover	Sklearn feature importances_
TC	70.869	34.703	39.018	70.869
HDL-C	15.291	30.007	31.527	15.291
TG	13.839	35.290	29.455	13.839

Table S2. ESC multiclass classification metrics for LDL-C estimation methods.

Method	Accuracy	Macro Precision	Macro Recall	Macro F1	Macro Specificity	Weighted Precision	Weighted Recall	Weighted F1	Micro F1	Macro OvR_AUC_LD
XGBoost	0.66	0.60	0.52	0.55	0.92	0.66	0.66	0.65	0.66	0.89
SVR	0.66	0.61	0.51	0.54	0.92	0.66	0.66	0.65	0.66	0.88
Random Forest	0.64	0.58	0.51	0.53	0.92	0.64	0.64	0.64	0.64	0.88
Linear Regression	0.62	0.58	0.40	0.43	0.91	0.62	0.62	0.60	0.62	0.87
Martin Formula	0.64	0.56	0.48	0.49	0.91	0.62	0.64	0.62	0.64	0.85
Sampson Formula	0.57	0.52	0.49	0.49	0.91	0.61	0.57	0.58	0.57	0.86
Friedewald Formula	0.41	0.38	0.41	0.35	0.88	0.54	0.41	0.44	0.41	0.75

Table S3. Bootstrap 95% confidence intervals for MAE, RMSE, and ESC classification accuracy based on pooled out-of-fold predictions from 10-fold cross-validation (development cohort, N = 11,681).

Method	MAE (mg/dL)	RMSE (mg/dL)	ESC Accuracy
XGBoost	14.681 (14.420–14.940)	20.256 (19.685–20.883)	0.656 (0.647–0.664)
SVR	14.810 (14.534–15.091)	21.171 (20.459–21.939)	0.659 (0.650–0.668)
Random Forest	15.540 (15.275–15.805)	21.163 (20.550–21.903)	0.638 (0.629–0.647)
Linear Regression	17.769 (17.400–18.198)	27.988 (24.279–33.594)	0.618 (0.609–0.626)
Martin	17.914 (17.372–18.539)	35.911 (28.785–46.047)	0.636 (0.627–0.644)
Sampson	18.803 (18.368–19.331)	32.120 (25.698–41.283)	0.570 (0.562–0.580)
Friedewald	28.884 (28.351–29.500)	42.554 (36.914–51.082)	0.407 (0.398–0.416)

Values are point estimates with bootstrap 95% confidence intervals in parentheses (5,000 patient-level resamples with replacement). MAE, mean absolute error; RMSE, root mean square error; ESC, European Society of Cardiology LDL-C treatment-group categories. Table 2 reports mean ± SD across cross-validation folds; this table reports bootstrap intervals from pooled out-of-fold predictions.

Table S4. Mathematical definitions of evaluation metrics.

Metric	Formula	Purpose	Interpretation
**MAE**	$MAE = (1/n) \times \Sigma \mid y_i - \hat{y}_i \mid$	Average absolute deviation (mg/dL); primary accuracy indicator	Lower = better; reported as MAD in Results
**RMSE**	$RMSE = \sqrt{[(1/n) \times \Sigma (y_i - \hat{y}_i)^2]}$	Penalises large errors more than MAE	Lower = better; sensitive to outliers
**MAD**	$MAD = (1/n) \times \Sigma \mid y_i - \hat{y}_i \mid$	Mean absolute difference between estimated and directly measured LDL-C (mg/dL); numerically equivalent to MAE when computed on the same predictions	Lower = better; unit: mg/dL. In this study, MAD was calculated from pooled out-of-fold predictions across 10-fold cross-validation
**ME**	$ME = (1/n) \times \Sigma (\hat{y}_i - y_i)$	Mean Error, Systematic directional bias (mg/dL)	ME > 0: overestimation; ME < 0: underestimation
**Bias%**	$Bias\% = [(\bar{\hat{y}} - \bar{y}) / \bar{y}] \times 100$	Relative systematic deviation from reference mean	Expressed as %; positive = overestimation
**r**	$r = \Sigma(y_i-\bar{y})(\hat{y}_i-\bar{\hat{y}}) / \sqrt{[\Sigma(y_i-\bar{y})^2 \times \Sigma(\hat{y}_i-\bar{\hat{y}})^2]}$	Linear association strength between estimated and measured LDL-C	Range [−1, 1]; insensitive to constant offset
**R^2**	$R^2 = 1 - [\Sigma(y_i - \hat{y}_i)^2 / \Sigma(y_i - \bar{y})^2]$	Explained variance; penalises systematic bias	$R^2 < 0$ if predictions worse than sample mean
**ESC Accuracy**	$Accuracy = (1/n) \times \Sigma I[cat(\hat{y}_i) = cat(y_i)]$	Proportion correctly classified into ESC LDL-C risk categories (<55, 55–69, 70–99, 100–115, 116–189, ≥190 mg/dL)	Range [0, 1]; reported to 3 decimal places

Abbreviations: MAE, mean absolute error; RMSE, root mean square error; ME, mean error; r, Pearson correlation coefficient; R^2, coefficient of determination; ESC, European Society of Cardiology; I(·), indicator function; y_i, directly measured LDL-C; $\hat{y}_i$, estimated LDL-C.

Table S5. Comparative Performance of LDL-C Estimation Methods at High Triglyceride Levels (>400 mg/dL)

Method	TG range	N	Predicted LDL ± mean_SD	MAE mg/dL	Mean_Error mg/dL	Bias %	r
Direct LDL-C	400-499 mg/dL	5857	123.10 ± 43.45	NA	NA	NA	NA
XGBoost			123.00 ± 38.35	14.77	-0.1	-0.1	0.89
SVR			124.77 ± 38.25	14.96	1.67	1.4	0.87
Random Forest			122.93 ± 38.77	15.64	-0.17	-0.1	0.87
Linear Regression			123.09 ± 31.84	17.68	-0.01	0	0.82
Martin			133.60 ± 44.84	17.73	10.5	8.5	0.79
Sampson			113.05 ± 39.22	18.6	-10.05	-8.2	0.83
Friedewald			100.40 ± 51.89	28.68	-22.69	-18.4	0.81
Direct LDL-C	500-599 mg/dL	2505	123.51 ± 41.74	NA	NA	NA	NA
XGBoost			123.81 ± 38.54	14.34	0.3	0.2	0.87
SVR			125.09 ± 37.91	14.23	1.58	1.3	0.88
Random Forest			123.56 ± 39.22	15.16	0.05	0	0.86
Linear Regression			124.03 ± 41.96	17.93	0.52	0.4	0.62
Martin			134.24 ± 60.51	18.45	10.73	8.7	0.57
Sampson			114.12 ± 55.41	19.3	-9.38	-7.6	0.58
Friedewald			101.65 ± 66.50	29.6	-21.86	-17.7	0.62
Direct LDL-C	600-699 mg/dL	1176	122.52 ± 43.10	NA	NA	NA	NA
XGBoost			122.66 ± 37.71	14.69	0.14	0.1	0.89
SVR			124.59 ± 37.57	15.11	2.07	1.7	0.86
Random Forest			122.61 ± 38.40	15.43	0.08	0.1	0.87
Linear Regression			123.04 ± 31.64	18.19	0.51	0.4	0.78
Martin			133.62 ± 45.22	18.5	11.1	9.1	0.74
Sampson			113.13 ± 39.22	19.17	-9.4	-7.7	0.78
Friedewald			100.65 ± 51.88	29.08	-21.87	-17.9	0.76
Direct LDL-C	700-799 mg/dL	653	124.83 ± 45.31	NA	NA	NA	NA
XGBoost			123.95 ± 41.30	14.97	-0.88	-0.7	0.89
SVR			125.11 ± 40.35	14.97	0.28	0.2	0.88
Random Forest			123.94 ± 41.43	16.15	-0.89	-0.7	0.88
Linear Regression			122.81 ± 33.42	17.12	-2.03	-1.6	0.86
Martin			133.56 ± 46.01	17.1	8.73	7	0.85
Sampson			113.28 ± 41.13	19.12	-11.55	-9.3	0.87
Friedewald			100.19 ± 54.20	30.25	-24.64	-19.7	0.86
Direct LDL-C	800-899 mg/dL	320	124.80 ± 40.91	NA	NA	NA	NA
XGBoost			123.07 ± 34.60	13.98	-1.73	-1.4	0.85
SVR			124.61 ± 34.53	13.86	-0.19	-0.2	0.84
Random Forest			122.49 ± 36.00	15.06	-2.31	-1.9	0.87
Linear Regression			123.52 ± 28.41	16.79	-1.28	-1	0.84
Martin			134.06 ± 40.75	15.67	9.26	7.4	0.83
Sampson			113.85 ± 34.67	17.93	-10.95	-8.8	0.86
Friedewald			102.39 ± 45.62	27.36	-22.41	-18	0.84

Direct LDL-C			126.83 ± 44.06	NA	NA	NA	NA
XGBoost			126.93 ± 37.10	15.74	0.1	0.1	0.87
SVR			128.24 ± 36.82	15.87	1.41	1.1	0.84
Random Forest			127.33 ± 39.10	16.51	0.5	0.4	0.87
Linear Regression			126.29 ± 30.56	17.83	-0.54	-0.4	0.86
Martin			137.59 ± 42.55	17.49	10.76	8.5	0.86
Sampson			116.66 ± 37.27	18.54	-10.17	-8	0.87
Friedewald	900-999 mg/dL	223	105.27 ± 49.26	27.5	-21.57	-17	0.87

Table S6. Baseline demographic and biochemical characteristics of the development and external validation cohorts.

Characteristic	Development	Roche	Beckman	Siemens
N analyzed	11,681	2,957	3,986	18,244
Age (years)	50.3 ± 12.7	46.2 ± 10.5	52.2 ± 11.8	47.1 ± 10.2
Sex - Male	7028 (60.2%)	1954 (66.1%)	2555 (64.1%)	11134 (61.0%)
Total cholesterol (mg/dL)	230 (199–265)	234 (202–272)	250 (215–290)	234 (203–269)
Triglycerides (mg/dL)	476 (425–570)	506 (441–648)	500 (438–624)	494 (437–614)
HDL-C (mg/dL)	32.0 (28.0–38.0)	32.5 ± 8.27	44.0 (38.0–52.0)	30.8 (26.0–36.5)
non-HDL-C (mg/dL)	197 (166–230)	201 (170–238)	205 (174–240)	202 (173–235)
LDL-C direct (mg/dL)	123 ± 43.1	119 ± 43.9	159 ± 40.8	114 (90.0–143)
Remnant cholesterol (mg/dL)	72.0 (56.0–92.0)	74.4 (57.4–103)	42.0 (31.0–58.0)	80.6 (63.8–104)

Variables are reported as mean ± SD or median (IQR) according to distribution.
